# Candida meningitis in three patients who underwent transsphenoidal surgery from a single-institution case series

**DOI:** 10.1007/s00701-026-06835-1

**Published:** 2026-03-13

**Authors:** Mattia Russel Pantalone, Francesca De Luca, Lovisa Terling, Alia Shamikh, Simon Skyrman, Martin Olsson

**Affiliations:** 1 Department of Neurosurgery, Karolinska University Hospital, Stockholm, Sweden; 2 Deparmtent of Medicine, Solna, Karolinska Institutet, Stockholm, Sweden; 3 Department of Neuroradiology, Karolinska University Hospital, Stockholm, Sweden; 4 Department of Pathology, Karolinska University Hospital, Stockholm, Sweden

**Keywords:** Candida meningitis, Transsphenoidal surgery, Craniopharyngioma

## Abstract

**Purpose:**

Candida meningitis is a rare but potentially deadly complication in neurosurgical patients. Most of the published case series include patients who underwent a craniotomy, while just three cases have been reported in patients operated on by transsphenoidal surgery.

**Methods:**

We performed an institutional review of patients who underwent transsphenoidal surgery over the last 5 years (2020–2024) at Karolinska University Hospital, Stockholm, Sweden, and searched for patients who were also diagnosed with Candida meningitis as a postoperative complication.

**Results:**

Out of over 400 operated patients, we identified three patients who were affected by postoperative candida meningitis. Two were male, (31 and 70 years old), and one female (37 years old). Pathological analyses revealed craniopharyngioma for the male patients and adenoma for the female patient. Postoperative CSF rhinorrhea occurred in all three patients, and they underwent endonasal endoscopic CSF leak repair surgery and were also treated with lumbar drainage. While none of them was previously immunocompromised, they all developed pituitary failure and were treated with hydrocortisone. The patients were successfully treated with Amphotericin B and Fluconazole.

**Conclusions:**

Although uncommon, Candida meningitis can occur in patients undergoing transsphenoidal surgery and should be suspected in cases of meningitis that do not respond to antibacterial drugs. Previous reports identified extremes of age and previous diagnosis of cancer and immunosuppression as risk factors while the three cases that we report here suggest that Candida meningitis can occur even in previously relatively healthy individuals. Other relevant risk factors for the development of Candida meningitis, such as large central tumors, postoperative CSF leakage, and prolonged cortisone use, should also be considered in the diagnostic process.

**Supplementary Information:**

The online version contains supplementary material available at 10.1007/s00701-026-06835-1.

## Introduction

Candida meningitis is a rare but severe fungal infection of the central nervous system (CNS), often associated with significant morbidity and mortality. It most commonly occurs in immunocompromised individuals, including patients with HIV, malignancies, or those receiving prolonged corticosteroid therapy. Clinically, Candida meningitis presents with nonspecific symptoms, such as fever, headache, altered mental status, and nuchal rigidity, often accompanied by nonspecific CSF findings. Due to its subtle and variable presentation, it is frequently misdiagnosed as bacterial or viral meningitis, resulting in delayed initiation of appropriate antifungal treatment and possible underdiagnosis. Candida meningitis is commonly fatal, especially in fragile individual already under antibiotic treatment, both to neurological compromission due to the infection. [8]. Co-infection with bacterial pathogens has also been described and in particular long term antibiotic treatment with consequent Clostridium difficile infection has been associated with higher incidence and mortality of Candida infection [12].

Candida meningitis has been reported following neurosurgical interventions in both immunocompromised and immunocompetent patients, particularly in those with CNS implants such as ventricular shunts or external ventricular drains (EVDs), as well as in trauma patients [2–4, 10, 11, 13]. Cases have also been documented after elective craniotomy; however, only three cases following transsphenoidal surgery have been reported to date [1].

Transsphenoidal surgery is a widely accepted minimally invasive approach for the resection of pituitary adenomas and other sellar or parasellar lesions. Despite its advantages, the approach traverses the sinonasal cavity, a known reservoir for fungal organisms, including Candida species [5]. Disruption of normal anatomical barriers and the occurrence of postoperative CSF leakage may increase the risk of secondary CNS infections, including fungal meningitis.

We conducted a retrospective institutional review of all patients who underwent transsphenoidal surgery at Karolinska University Hospital (Stockholm, Sweden) between 2020 and 2024 to determine the incidence of postoperative Candida meningitis.

Among 410 operated patients, three were diagnosed with Candida meningitis postoperatively. Here, we present these clinical cases in detail and compare their characteristics and outcomes with those reported in the existing literature.

## Case 1

**Fig. 1:**
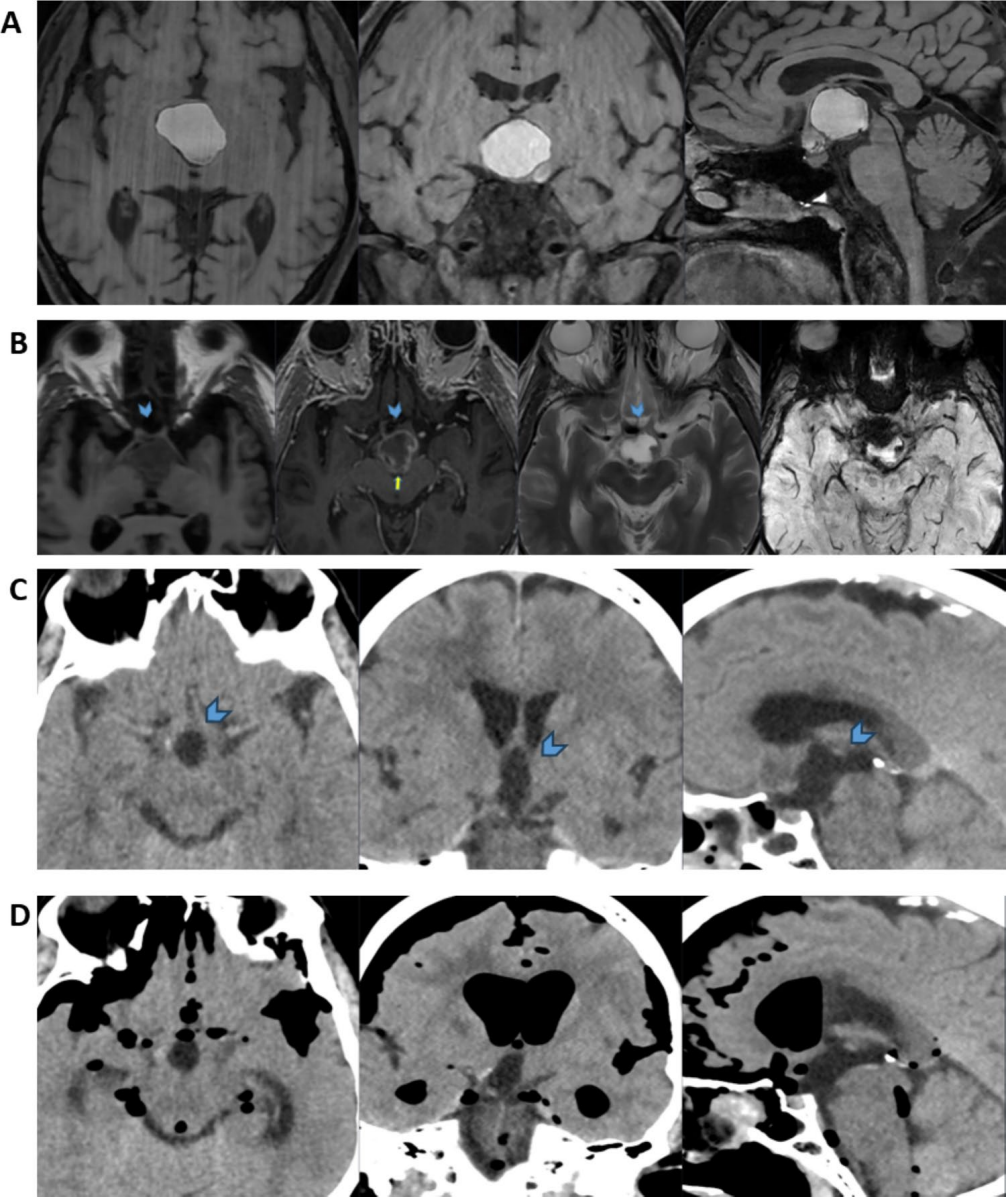
**A**) Native T1-w, axial, coronal, and sagittal views. Large predominantly cystic suprasellar expansiveness. The lesion is located in the suprasellar cistern, as appreciated in the sagittal view. Native high signal on T1-w is seen, suggestive of a significant intra-lesional subacute bleeding. **B**) Axial native T1-w, CE T1-w, T2-w, and SWI. MRI post endoscopic fenestration of cystic craniopharyngioma, via a right frontal approach. Compared to the preoperative MRI dated 2023–08–09, a centimeter-sized reduction of the cyst in all axes is appreciated. Slight edema and minor amounts of postoperative blood and small amounts of intraventricular air. The contrast enhancement in the cyst wall was seen as slightly increased compared to previous imaging (yellow arrow), and interpreted as postoperative-related changes. The optic chiasm is located anteriorly and is elevated by the cyst (blue arrowhead). **C**) Axial, coronal, and sagittal brain CT, soft-tissue window. Post resection status, no evident residual tumor, and no major bleeding (blue arrowhead). **D**) Onset of extensive supra- and infratentorial pneumocephalus, including intraventricular air causing hydrocephalus. High-attenuating hematoma posteriorly in the ethmoidal sinus (yellow arrow).

A 70-year-old man with no prior comorbidities presented in summer 2023 with fatigue, visual impairment, and weight gain. MRI revealed a suprasellar cystic lesion consistent with a craniopharyngioma (Fig. 1A). The mass was near the foramen of Monro, and an initial endoscopic cyst fenestration was performed to prevent obstructive hydrocephalus. Postoperatively, the patient remained stable, and MRI confirmed cyst reduction without hemorrhage (Fig. 1B). Pituitary function was intact, and no hormonal substitution was required.

Ten days later, transsphenoidal tumor extirpation was performed. The infundibulum appeared infiltrated and was removed for gross total resection. The surgical closure included autologous fat, fascia, and a Hadad flap. No intraoperative CSF leakage was noted. A lumbar drain was placed postoperatively. CT showed no residual tumor, hemorrhage, or infarction (Fig. 1C). Two days postoperatively, the patient developed diabetes insipidus and adrenal insufficiency, treated with desmopressin and hydrocortisone. Pathology confirmed adamantinomatous craniopharyngioma (WHO grade I, Figure S1).

Two weeks after surgery, the patient presented with epistaxis, headache, neck stiffness, and reduced consciousness (GCS 13). CT showed pneumocephalus (Fig. 1D). CSF analysis revealed pleocytosis (≈ 500 cells/μL, polymorphonuclear predominance), elevated lactate (10.8 mmol/L), low CSF-glucose (CSF/P ratio 0.13), and elevated CSF albumin (8,076 mg/L). Empiric treatment with linezolid (600 mg × 2) and meropenem (2 g × 3) was started, and the hydrocortisone dose was increased. Revision surgery for postoperative leakage was performed three days later.

**Fig. 2:**
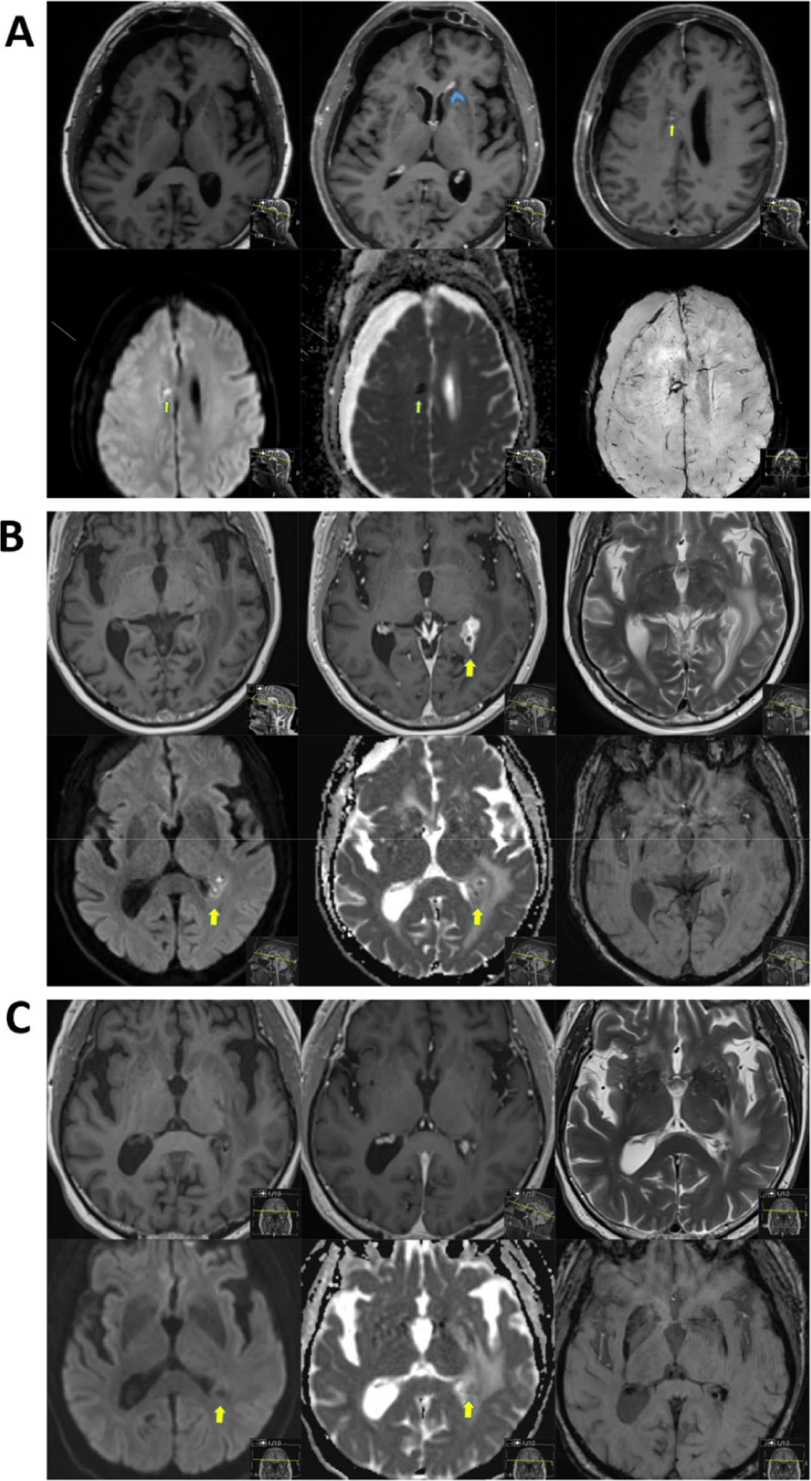
**A**) Axial native T1-w, CE T1-w (upper row), DWI, ADC, and SWI (lower row). Signs of meningitis with ventriculitis (blue arrowhead) as well as a suspected small periventricular abscess formation with signs of restricted diffusion (yellow arrow). Mass effect from a right-sided subdural hematoma, otherwise no obvious signs of increased intracranial pressure. **B**) Axial native T1-w, CE T1-w (upper row), DWI, ADC, and SWI (lower row). Clustered small intracranial abscesses near the left trigone with obstruction of the left temporal horn and ventriculitis (yellow arrow). The previously described right-sided subdural hematoma appears now subdivided into multiple compartments, likely with small portions of empyema (not shown in the image). Mild hydrocephalus is seen. **C**) Axial native T1-w, CE T1-w (upper row), DWI, ADC, and SWI (lower row). Regression of ventriculitis and abscesses is observed. Imaging shows no longer signs of restricted diffusion (yellow arrow), indicating an absence of active infection.

The patient later developed CSF rhinorrhea, headache, and seizures. CSF again showed inflammatory findings and PCR positivity for HSV-1. Cultures from nasal tamponades grew E. coli and yeasts, prompting treatment with meropenem and ampicillin. MRI demonstrated meningitis, ventriculitis, and a periventricular abscess (Fig. 2A). Candida albicans grew in CSF culture, though β-glucan was negative. Given poor clinical response, combination therapy with amphotericin B and flucytosine was initiated. After a few days of the combined treatment, the patient developed C. difficile enteritis. After seven days, flucytosine was discontinued due to renal failure, and therapy was switched to oral fluconazole. Amphotericin B was continued for three weeks.

Despite improvement, follow-up MRI after one month showed progression of the left trigonal abscess (Fig. 2B). Stereotactic biopsy revealed reactive gliosis and granulomatous inflammation. Cultures were negative, but sequencing identified C. albicans DNA, confirming intracranial fungal infection. The patient continued fluconazole for nine months, achieving full clinical recovery (Fig. 2C). MRI control after one year (Fig. 2C) from extirpation surgery revealed no tumor recurrence and no abscess.

## Case 2

**Fig. 3:**
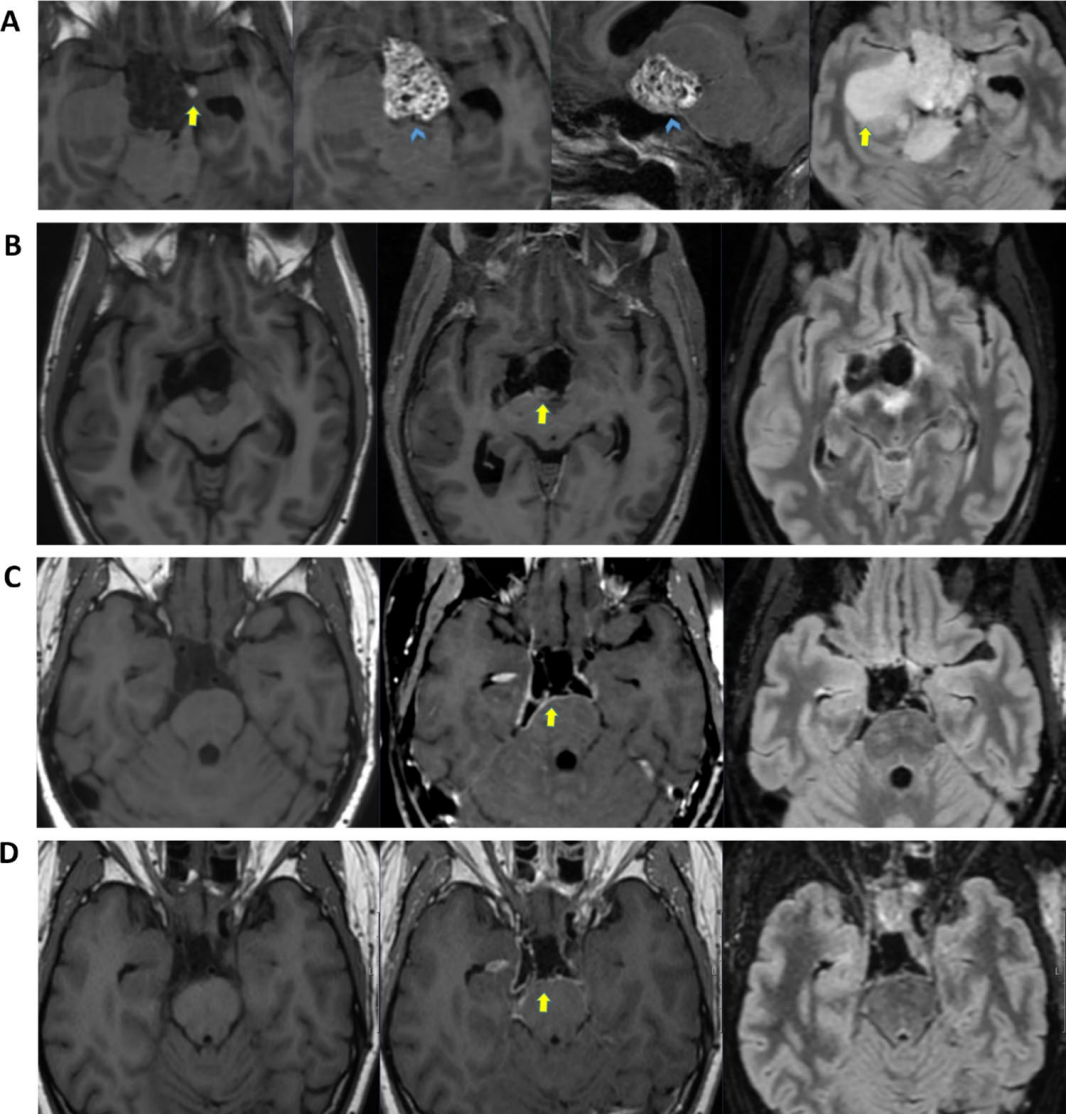
**A**) Axial native T1-w, CE T1-w, sagittal CE T1-w, and axial T2-FLAIR. A centrally located tumor with both calcified and cystic non-contrast-enhancing components (yellow arrow) and contrast-enhancing components (blue arrow-head), causing obstructive hydrocephalus. Craniopharyngioma was given as the top diagnostic differential. **B**) Axial native T1-w, CE T1-w, and axial T2-FLAIR. Good postoperative status with suspicion of only minimal residual tumor in the interpeduncular cistern (yellow arrow). **C**) Axial native T1-w, CE T1-w, and axial T2-FLAIR. Extra-axial contrast-enhancement along the resection margins was seen, of difficult interpretation if related to residual tumor-related changes or meningitis-related changes. Renewed MRI after meningitis treatment completion is recommended in the radiological report. **D**) Axial native T1-w, CE T1-w, and axial T2-FLAIR. Unchanged extra-axial contrast-enhancement compared to the previous MRI exam, no signs of restricted diffusion, thus no radiological suspicion of meningitis. Still, of difficult assessment whether the extra-axial contrast-enhancement is related to postoperative changes or minimal tumor recurrence/remnant tumor. Further follow-up with an MRI in 3–6 months is suggested in the radiological report for reassessment.

A 31-year-old man, previously healthy and with no regular medications, but with regular consumption of Kratom in the last year. The patient developed personality changes, fatigue, balance problems, apraxia, and bitemporal visual field loss in the autumn of 2024. MRI via primary care revealed a central tumor with a cystic component and obstructive hydrocephalus. The patient was admitted to the neurosurgery ward and bilateral ventricular drainage was inserted. A more comprehensive MR was then performed at our hospital (Fig. 3A). A week later, the patient underwent transsphenoidal tumor resection. MRI post op revealed minimal residual tumor (Fig. 3B). Pathological analyses revealed craniopharyngioma grade I (Figure S2). Postoperatively, the patient developed hypopituitarism and diabetes insipidus, and treatment with hydrocortisone, desmopressin, levothyroxine, and testosterone was started. CSF testing revealed rising lactate and leukocytosis with poly predominance, although no bacterial growth was observed in culture. Treatment for suspected meningitis was initiated with linezolid and meropenem. Clinically, the patient did not show other systemic signs of infection with low C-reactive protein and no fever, clinical and radiological improvement of ventricular dimension. Therefore, the ventricle drainages were discontinued, and one week after surgery, the patient was transferred to the endocrinology ward for continued adjustment of fluid balance. However, just three days later, the patient developed CSF rhinorrhea and was transferred again to the neurosurgery department. The patient received a lumbar drainage and was operated with endoscopic endonasal CSF leak repair surgery.

Despite antibiotic treatment, no clinical improved was observed after two weeks of treatment. No bacterial growth was observed, and sequencing did not reveal bacterial DNA.

CSF culture showed growth of C. albicans and despite the initial assessment that it could be a contaminant, beta-glucan turned out positive, and after 5 days form positive culture, treatment with amphotericin B, flucytosine, and fluconazole was initiated. Amfotericin B and flucytosine treatment continued for 3 weeks, while fluconazole for 6 months. Interestingly, the patient had also developed oral and cutaneous candidosis.

Additional testing revealed Staphylococcus epidermidis and Propionibacterium growth in CSF culture. This was possibly associated to repeated leakage from the lumbar drain with multiple replacements. An additional sealing surgery was performed because of recurrent leakage.

The patient was deemed to require permanent cerebrospinal fluid drainage.

The patient continued antifungal treatment in the neurosurgical ward, with gradual improvement in cerebrospinal fluid results with decreasing beta-glucane, lactate, and leukocyte (poly predominance). After two weeks of antifungal treatment and repeated CSF analyses showing decreased cells and beta-glucan levels, a ventriculoperitoneal (VP) shunt (Strata valve, regular model, Medtronic) was inserted.

The patient clinically improved. A lumbar puncture ten days after VP-shunt showed decreasing beta-glucan levels and no growth in culture. The patient was then transferred to a specialized rehabilitation center.

MRI brain control 3 months after surgery (Fig. 3C) showed still signs of contrast enhancement related to meningitis/ventriculitis and minimal residual tumor.

MRI brain control after 6 months (Fig. 3D) showed no sign of meningitis and no residual tumor.

## Case 3

A 37-year-old woman with depression, pronounced iatrophobia, and obesity (BMI 40.5) was found unresponsive at home in August 2024 and admitted to the emergency department. Blood tests revealed severe diabetic ketoacidosis (pH 6.6) and hypokalemia (2.2 mmol/L). She required three days in the ICU with intravenous fluids, potassium, and continuous insulin infusion. Empiric intravenous cloxacillin and cefotaxime were administered for a concomitant right axillary infection. New-onset diabetes was confirmed (HbA1c 148 mmol/mol) with marked insulin resistance. Additional findings included clinical signs of acromegaly (coarse facial features, large hands and feet), dilated cardiomyopathy with reduced global systolic function (LVEF 45–50%), and bilateral radial artery thromboses related to arterial cannulation; apixaban (Eliquis) was initiated. Fungal infection in the genital area was also noted.

**Fig. 4:**
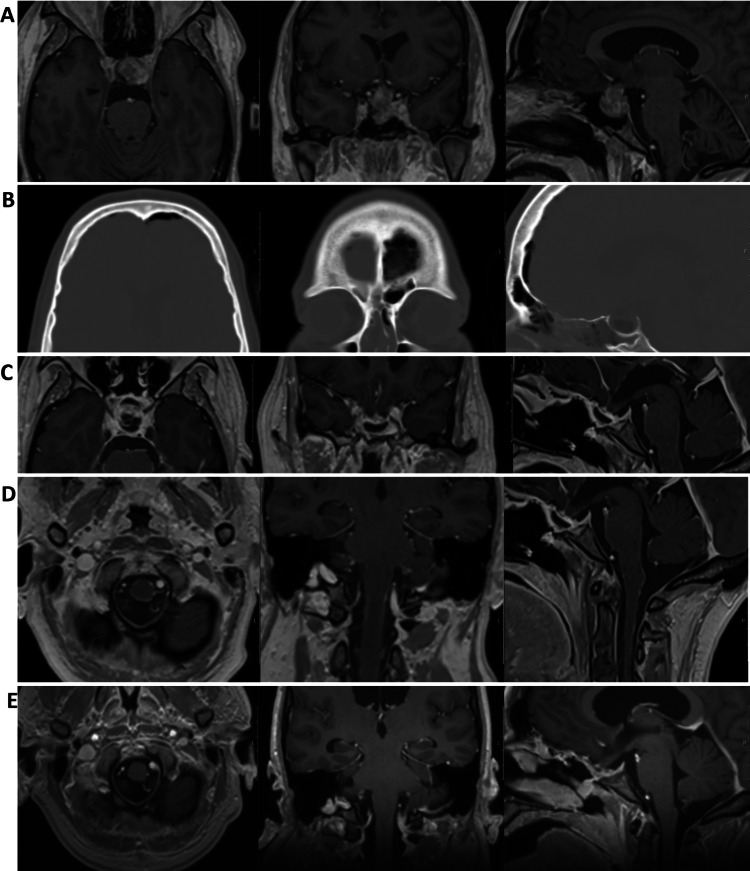
**A**) CE T1-w axial, coronal, and sagittal MRI brain images. A 2.5 cm large suspected macroadenoma engaging the hypophyseal stalk with compression of the optic chiasm and parasellar extension. **B**) Axial, coronal, and sagittal CT brain, bone window. Post-resection status, small amount of pneumocephalus. Fat grafting has been performed at the tumor site and within the sphenoidal sinus (sagittal view). **C**, **D**) CE T1-w axial, coronal, and sagittal MRI brain images. Signs of extensive leptomeningitis in the sellar and infratentorial (C) and in the infratentorial and spinal region (D). Appearance consistent with adhesions in the sella, deformed suprasellar structures, and edema in the markedly displaced optic chiasm. No definitive signs of cerebrospinal fluid leakage were observed. **E**) CE T1-w axial, coronal, and sagittal MRI brain images. Partial regression of leptomeningeal contrast-enhancement in the infratentorial and spinal region. Unchanged postoperative status in the sellar region with displacement of the optic chiasm

Pituitary MRI demonstrated a 21 × 21 × 23 mm macroadenoma with suprasellar extension elevating the optic chiasm (Fig. 4A). Ophthalmologic assessment showed preserved visual acuity. Endocrine tests revealed low TSH, high GH, and subsequently elevated IGF-1, consistent with active acromegaly. Interestingly, IGF-1 was initially within the reference range, possibly due to the severe catabolic state. The patient underwent endoscopic endonasal transsphenoidal resection in the second half of September 2024. Perioperatively, a high-flow CSF leak occurred. Pathology confirmed a PitNET/adenoma with focal GH and prolactin expression (Ki-67 1.3%, focally up to 3.7%) (Figure S3).

Postoperatively, she developed a CSF leak confirmed by β-trace testing. Cefuroxime was initiated, and a lumbar drain was inserted. The patient developed headache and photophobia, and bacterial meningitis was confirmed with growth of Proteus mirabilis in CSF culture. CT imaging revealed small intracranial air, no major bleeding, and satisfactory tumor resection (Fig. 4B). She was then treated with IV meropenem and linezolid. Persistent CSF leakage required placement of an external ventricular drain and two additional endoscopic leak-repair surgeries on 30 September and 10 October. No further leakage occurred thereafter, and she was discharged one week later.

In November–December 2024, she experienced several readmissions for fever and malaise. In early November, a urine culture again grew Proteus mirabilis without clear urinary tract symptoms; recurrent bacterial meningitis was considered unlikely. During the first two weeks of December, she was readmitted twice with fever, chills, and severe ear pain. Blood tests showed low CRP. Lumbar puncture revealed inflammatory CSF with leukocytosis (polymorphonuclear predominance) and elevated lactate, but no bacterial growth. These episodes were managed as non-pyogenic (“chemical”) meningitis related to prior surgery and CSF leakage, with symptomatic treatment.

At the beginning of January 2025, she was admitted to the infectious diseases ward. MRI showed leptomeningeal contrast enhancement (Figs. 4C and D). Broad CSF testing, including sequencing, detected Candida albicans DNA, consistent with fungal meningitis. Intravenous liposomal amphotericin B (AmBisome) was initiated in February and continued for six weeks, resulting in clear clinical improvement, after which she was transitioned to oral fluconazole. Shortly thereafter (April), she deteriorated with critically prolonged QTc (peak 658 ms), hypercalcemia, hypokalemia, adrenal insufficiency requiring hydrocortisone, and moderate protein-energy malnutrition. Fluconazole was discontinued, IV liposomal amphotericin B was reintroduced, electrolytes were corrected, and nutritional support was provided, leading to subsequent stabilization.

At follow-up at the end of June 2025, she reported progressive improvement with restored energy and resolution of chronic pain. On OGTT, GH was appropriately suppressed; IGF-1 remained moderately elevated, and a watch-and-wait strategy was chosen before initiating medical therapy. She remained on long-term oral fluconazole with close monitoring. The most recent lumbar puncture (mid-June 2025) showed decreasing CSF inflammation and no detectable Candida DNA. Follow-up MRI demonstrated no residual tumor, decreased leptomeningeal contrast enhancement (Fig. 4E), and stable mild–moderate hydrocephalus attributed to prior meningitis. No CSF shunt was indicated, given her clinical course. Cardiac function had improved, diabetes was well controlled on sitagliptin, and she continued temporary low-dose hydrocortisone.

## Discussion

In this institutional review of transsphenoidal surgery cases performed over the past five years at the Department of Neurosurgery, we identified three patients who developed Candida meningitis as a postoperative complication. To our knowledge, only three similar cases have previously been reported in the literature by Bridges and colleagues [1]. In their study of almost 400 patients, three cases of postoperative Candida meningitis were observed: two men and one woman. Although Candida infections may be underdiagnosed, our data and that of Bridges et al. suggest a comparable incidence of less than 1%.

Given this low incidence, it remains challenging to define specific risk factors for this complication in the context of transsphenoidal surgery. A limitation of our study is that just three patients were diagnosed with the condition, and therefore, it may still be challenging to define guidelines that may be broadly applied in clinical practice. However, several common features were observed among the reported patients. All six patients (three in our cohort and three from Bridges et al.) received hydrocortisone supplementation; five of the six developed postoperative CSF rhinorrhea requiring repair surgery. All patients had an external ventricular drain and a history of antecedent bacterial infection treated with broad-spectrum antibiotics. In this regard, we can speculate that reduced intracranial pressure related to cerebrospinal fluid diversion may influence the integrity of skull base reconstruction. Lumbar drainage or ventricular drainage can initially reduce the effectiveness of closure techniques by masking or perpetuating a minimal communication between the extracranial and intracranial spaces, even before overt cerebrospinal fluid leakage becomes clinically evident. Such occult or intermittent CSF fistulas may therefore precede the onset of infection and contribute to the development of postoperative meningitis. In this context, the indication for postoperative CSF diversion should be carefully weighed against its potential risks, and a tailored, case-by-case risk–benefit assessment is warranted, particularly in patients with persistent infection or repeated repair procedures.

While the three cases reported by Bridges et al. involved different Candida species (C. glabrata, C. tropicalis, and C. albicans), C. albicans was identified in all three of our cases. Interestingly, two of our patients also exhibited concurrent cutaneous fungal infections. None of our patients had a documented history of chronic use of topical nasal corticosteroids or decongestants before surgery. At our institution, all patients undergoing endoscopic transsphenoidal surgery routinely receive preoperative MRI and CT of the skull base, including assessment of the sphenoid sinus anatomy. None of the patients in this series demonstrated radiological signs of acute or chronic invasive fungal sinus disease preoperatively.

One of our three patients (Case 3) had a high BMI of 40.5, a known risk factor for postoperative CSF leak [6, 9]. This patient also suffered from iatrophobia, which may have delayed the initial diagnostic process. None of our patients developed candidemia. In contrast, the only fatal case described in the literature was associated with vasculitis and rupture of a mycotic basilar artery aneurysm; that patient had previously undergone abdominal surgery, which has been shown to increase the risk of Candida infection. None of our patients had abdominal operations recently.

In our department, cerebrospinal fluid analysis is routinely performed twice weekly in patients with external ventricular drainage. In our cases, initial results suggested bacterial meningitis. A key takeaway is that fungal meningitis should be promptly suspected in neurosurgical patients showing inadequate response to antibiotic treatment, especially those who are immunosuppressed. Candida should not be readily dismissed as a culture contaminant but rather considered a relevant differential diagnosis. This should lead to an implementation of institutional protocols with routine testing for fungal infection in CSF in patients with long-term antibiotic treatment to promptly treat this condition.

Currently, no pre-emptive antifungal therapy is included in standard treatment protocols for adult patients, but we believe it may be reasonable to consider prophylaxis in selected high-risk cases. Larger studies are needed to validate this approach. For example, nystatin “swish-and-swallow” prophylaxis has been shown to reduce fungal colonization in surgical and trauma ICU patients [7].

## Conclusion

While Candida meningitis remains an uncommon complication of neurosurgical procedures, including transsphenoidal surgery, its recognition is crucial due to its potentially fatal consequences. A high index of suspicion should be maintained in post-neurosurgical patients presenting with meningitic symptoms, particularly those with prolonged antibiotic or corticosteroid treatment, CSF leaks, or external drains. Early diagnosis, guided by CSF culture and fungal biomarkers, and timely antifungal therapy are critical to improve patient outcomes.

Future studies and larger case series are needed to better understand the risk factors and refine preventive strategies to minimize fungal CNS infections in neurosurgical patients.

## Supplementary Information

Below is the link to the electronic supplementary material.

Supplementary file1 (DOCX 10872 KB)

## Data Availability

No datasets were generated or analysed during the current study.

## Abbreviations

CNS: Central nervous system
CSF: Cerebrospinal fluid
CT: Computed tomography
ICU: Intensive care unit
MRI: Magnetic resonance imaging
