# Supplementary material for: Candida meningitis in three patients who underwent transsphenoidal surgery from a single-institution case series
Source: Acta Neurochir (Wien). 2026 Mar 13;168(1):71. doi: 10.1007/s00701-026-06835-1 (PMC12988994; doi:10.1007/s00701-026-06835-1)
Supplement: Supplementary file 1 — Supplementary file1 (DOCX 10872 KB) [file 701_2026_6835_MOESM1_ESM.docx]

**Candida meningitis in three patients who underwent transsphenoidal surgery from a single-institution case series.**


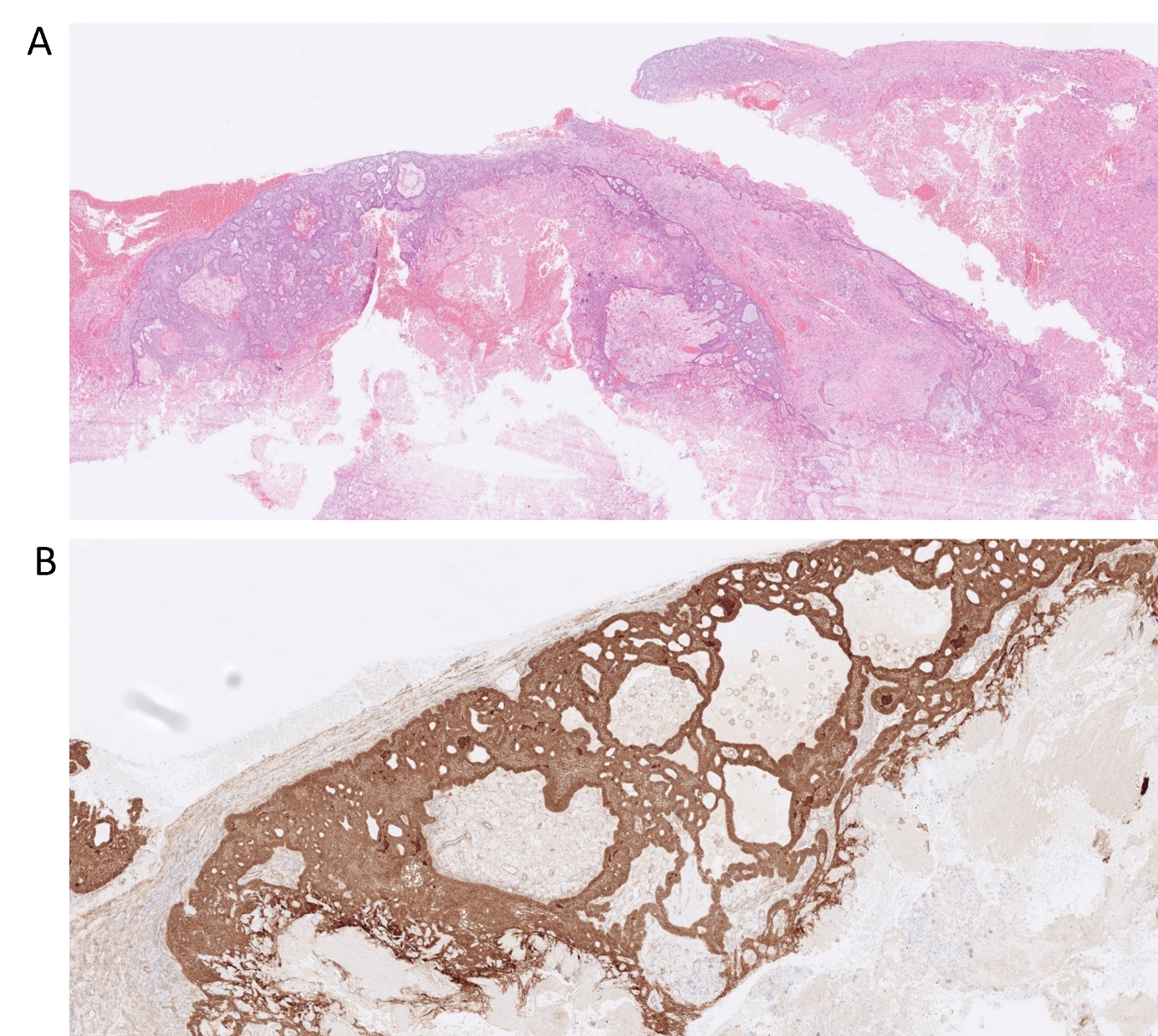


**Figure S1**

A) Epithelial cells with peripheral palisading and central stellate stroma are seen, in places with so-called "wet keratin" and calcifications.

B) Supplementary immunohistochemical analysis shows beta-catenin accumulation in a subpopulation of the epithelial cells, consistent with activation of the WNT signaling pathway.

Overall findings are consistent with adamantinomatous craniopharyngioma, CNS WHO grade 1.


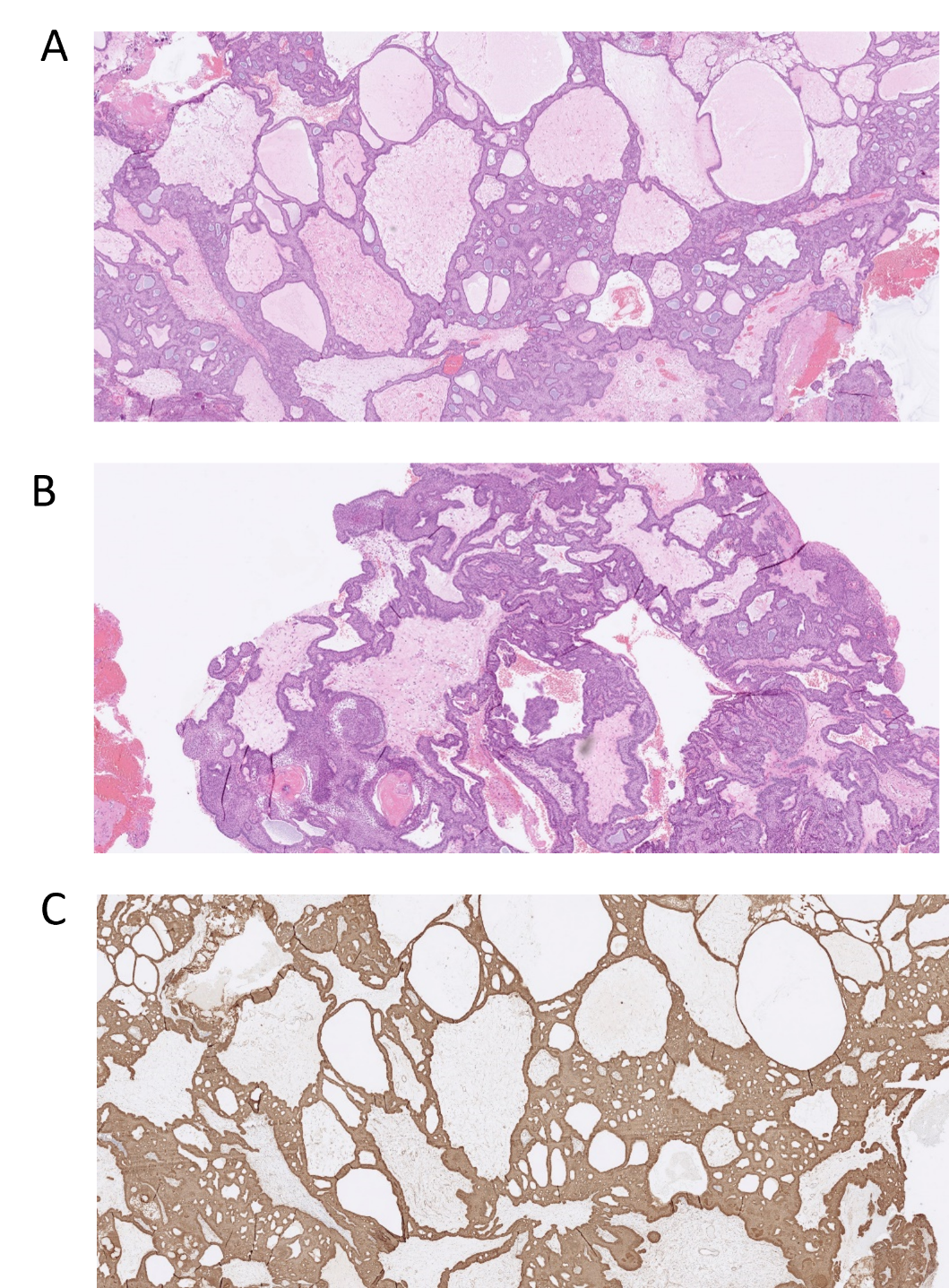


**Figure S2**

A, B) Strands of epithelial cells with peripheral palisading and central stellate stroma are seen, in places with so-called "wet keratin" and calcifications.

C) Immunohistochemical analysis shows focal nuclear accumulation of beta-catenin, consistent with activation of the WNT signaling pathway/CTNNB1 mutation.

Overall findings are consistent with adamantinomatous craniopharyngioma, CNS WHO grade 1.


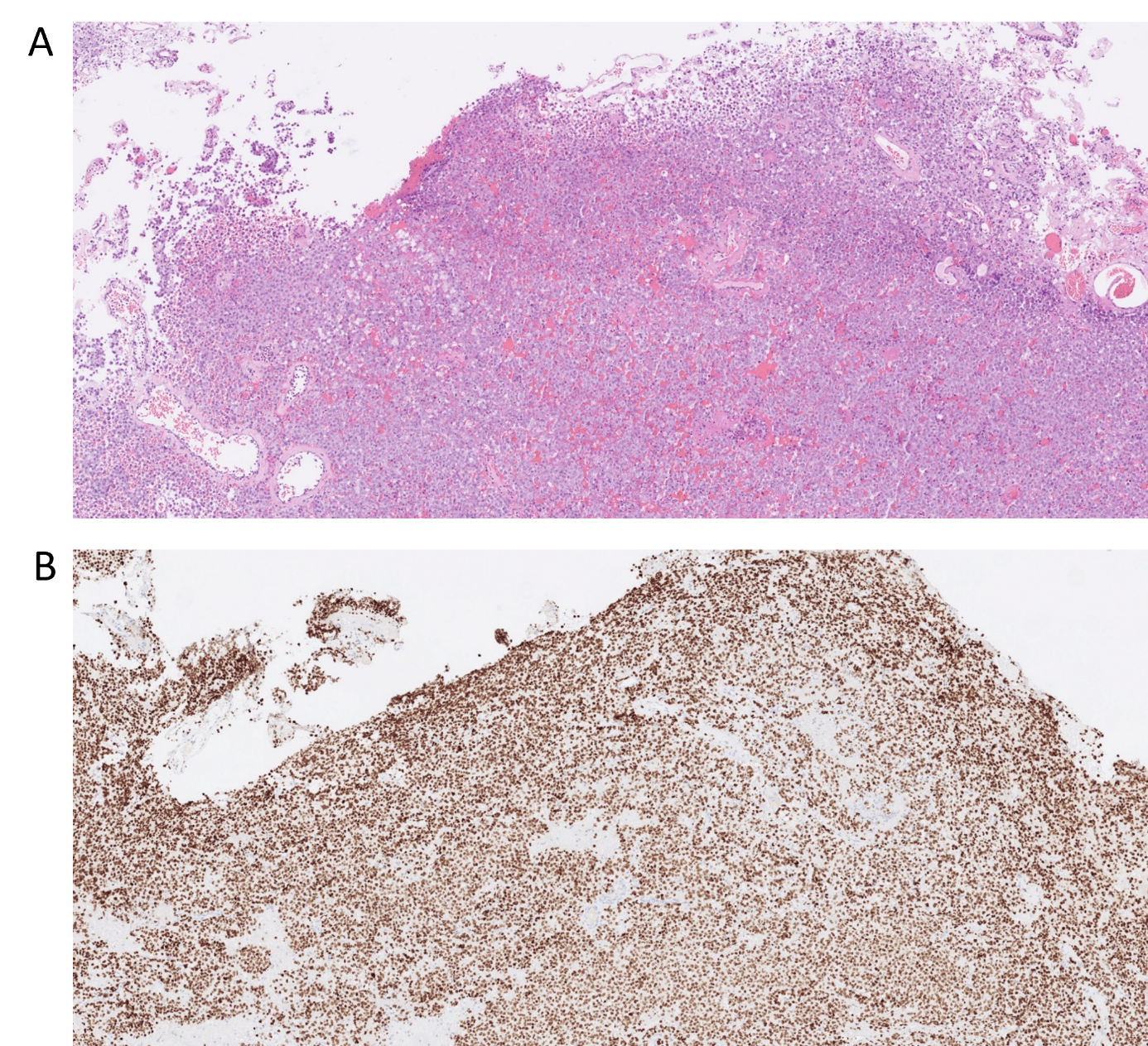


**Figure S3**

A) Microscopically, a compact proliferation of polygonal epithelial cells is seen, with a moderate amount of pale, vesicular cytoplasm and irregularly shaped nuclei showing open chromatin and distinct nucleoli. Occasional cells display larger, pleomorphic nuclei and suggest intranuclear pseudo-inclusions. Mitotic figures are scarce.

B) PitNET/adenoma with diffuse expression of PIT1 and focal expression of GH and prolactin, most consistent with an immature PIT1-lineage PitNET/adenoma.
